# TTT and PIKK Complex Genes Reverted to Single Copy Following Polyploidization and Retain Function Despite Massive Retrotransposition in Maize

**DOI:** 10.3389/fpls.2017.01723

**Published:** 2017-11-07

**Authors:** Nelson Garcia, Joachim Messing

**Affiliations:** Waksman Institute of Microbiology, Rutgers University, Piscataway, NJ, United States

**Keywords:** gene balance hypothesis, TTT complex, PIKK, genome fractionation, gene body methylation

## Abstract

The TEL2, TTI1, and TTI2 proteins are co-chaperones for heat shock protein 90 (HSP90) to regulate the protein folding and maturation of phosphatidylinositol 3-kinase-related kinases (PIKKs). Referred to as the TTT complex, the genes that encode them are highly conserved from man to maize. TTT complex and PIKK genes exist mostly as single copy genes in organisms where they have been characterized. Members of this interacting protein network in maize were identified and synteny analyses were performed to study their evolution. Similar to other species, there is only one copy of each of these genes in maize which was due to a loss of the duplicated copy created by ancient allotetraploidy. Moreover, the retained copies of the TTT complex and the PIKK genes tolerated extensive retrotransposon insertion in their introns that resulted in increased gene lengths and gene body methylation, without apparent effect in normal gene expression and function. The results raise an interesting question on whether the reversion to single copy was due to selection against deleterious unbalanced gene duplications between members of the complex as predicted by the gene balance hypothesis, or due to neutral loss of extra copies. Uneven alteration of dosage either by adding extra copies or modulating gene expression of complex members is being proposed as a means to investigate whether the data supports the gene balance hypothesis or not.

## Introduction

Maize has undergone a whole-genome duplication event due to allotetraploidization approximately 4.8 million years ago (Swigonova et al., 2004) and was domesticated only about 10,000 years ago (Doebley, 2004). The whole-genome duplication event created 20 pairs of chromosomes, which were later reduced to 10 after diploidization, mostly by chromosome fusions and deletions of centromeres (Wei et al., 2007; Salse et al., 2008). The maize genome also underwent extensive retrotransposition, gene movement (Lai et al., 2004), chromosome contraction and fractionation, and the loss of homoeologous gene copies (Bruggmann et al., 2006; Schnable et al., 2011).

Several hypotheses explain this fractionation process. The gene balance hypothesis calls for an ideal range of stoichiometric balance between members of protein complexes because a disruption due to imbalance in concentrations of the components can have harmful effects (Birchler and Newton, 1981; Veitia, 2002; Papp et al., 2003). This dosage-dependent function can also apply to the interaction of positive and negative regulatory effectors (Birchler et al., 2005), and involved many genes of different functions (Birchler et al., 2001). Indeed, genes that are thought to be dose-sensitive such as those encoding components of proteasome/protein modification complexes, signal transduction machinery, ribosomes, and transcription factor complexes mendelized in *Arabidopsis* after diploidization from its tetraploid ancestor (Thomas et al., 2006). The same study used the term “connected genes” to describe loci that seemed to be co-regulated and dependent on each other. In addition, there seems to be preferential removal of genes from one of the homoeologs, the same as in maize (Woodhouse et al., 2010). This process is probably a common occurrence in eukaryotes with whole-genome duplication histories (Birchler et al., 2001; Sankoff et al., 2010). Preferential removal of genes from one homoeolog also explains the uneven expansion and contraction of syntenic blocks in maize (Bruggmann et al., 2006). On the other hand, loss of a duplicated copy could simply be due to neutral loss of an extra copy over evolutionary time.

Although genome-wide studies support the fractionation bias of connected genes, we thought to investigate this at the level of specific examples of interacting genes that are parts of functional units during development. We recently described the first TTT co-chaperone complex in plants (Garcia et al., 2017). The TTT complex is composed of Telomere maintenance 2 (Tel2), Tel2-interacting protein 1 (Tti1), and Tel2-interacting protein 2 (Tti2) and functions as co-chaperones for maturation and stability of phosphatidylinositol 3-kinase-related kinases (PIKKs) (Hurov et al., 2010; Takai et al., 2010). The PIKK family on the other hand is involved in cell signaling related to growth in response to nutrients (TOR), DNA damage response (ATM, ATR, and DNA-PKcs), epigenetic transcriptional regulation (TRRAP), and nonsense-mediated RNA decay (SMG-1) (Abraham, 2004; Lovejoy and Cortez, 2009). Mutations in the TTT complex and PIKK members are lethal in many organisms (Brown and Baltimore, 2000; Benard et al., 2001; Menand et al., 2002; Takai et al., 2007; Stirling et al., 2011; Yamamoto et al., 2012). Deregulated expression has been implicated in many diseases including cancer (Populo et al., 2012; Weber and Ryan, 2015; Rodina et al., 2016), which underscores the essential function of these proteins.

## Materials and Methods

The human TEL2 (UniProt Q9Y4R8), TTI1 (UniProt O43156), and TTI2 (UniProt Q6NXR4) protein sequences were used to identify orthologs in maize (version 3 assembly) and other animal and fungal species using BLASTP at default settings. These proteins are well-characterized in yeast and mammals and have unique conserved domains that can be used to identify orthologs. The selected maize sequences were then used to identify other plant TEL2, TTI1, and TTI2 homologs using a BLASTP search in the Phytozome database. Sequences from representative organisms were then selected and aligned using ClustalW. A maximum likelihood phylogenetic tree was then created using the JTT model as implemented in the software package MEGA 6 (Tamura et al., 2007), with 500 bootstrap replications to test for clade confidence. The accession numbers for the sequences used are listed in **Table 1**. For the PIKKs, *Arabidopsis* TOR (UniProt Q9FR53), ATM (UniProt Q9M3G7), ATR (UniProt Q9FKS4), and human DNA-PKcs (UniProt P78527), SMG-1 (UniProt Q96Q15), and TRRAP (UniProt Q9Y4A5) were used to find their orthologs in maize, sorghum, and rice using BLASTP at default settings. Maize syntenic homologs and their subgenome assignments were obtained from the sorghum-referenced Pan-Grass Syntenic Gene Set (Schnable et al., 2012, 2016). Transposable element (TE) insertions in introns were identified based on RepeatMasker annotations. Previous whole genome DNA methylation studies in B73 from West et al. (2014) were used to identify the CG, CHG, and CHH methylations of these TEs.

**Table 1:** Accession numbers used in phylogenetic analysis of TTT complex members.

Species	TEL2	TTI1	TTI2
*Homo sapiens*	NP_057195.2	NP_055472.1	NP_079391.2
*Pan troglodytes*	XP_016784658.1	XP_514634.2	XP_016814485.1
*Mus musculus*	NP_082156.2	NP_083558.1	NP_659176.2
*Danio rerio*	NP_001071209.1	XP_698602.6	XP_002663251.1
*Apis mellifera*	XP_006571695.1	XP_006562728.2	XP_006557328.1
*Zea mays*	GRMZM2G144166	GRMZM2G056403	GRMZM2G048851
*Oryza sativa*	LOC_Os02g38680	LOC_Os10g42510	LOC_Os06g47900
*Arabidopsis thaliana*	AT3G48470	AT2G39910	AT1G79190
*Solanum lycopersicum*	Solyc10g006780.2	Solyc12g017280.1	Solyc06g009690.1
*Glycine max*	Glyma.04G193000	Glyma.09G284900	Glyma.18G031000
*Saccharomyces cerevisiae*	NP_011613.3	NP_011613.3	NP_012670.3
*Gibberella zeae*	EYB30669.1	EYB26245.1	EYB24810.1
*Aspergillus oryzae*	XP_001821710.2	XP_001826375.1	KDE78635.1


To estimate the insertion time of the LTR-retrotransposons in maize, we used RepeatMasker to retrieve the left and right LTR sequences and aligned them using CLUSTALW. Nucleotide substitution rates between the two LTRs were then calculated using MEGA 6 software using the Distance Estimation analysis option with the Kimura 2-parameter method. Uniform rates were assumed among sites and gaps were deleted from the analysis. Insertion time was then calculated using the reported estimate for LTR nucleotide substitution rate of 1.3 × 10^-8^ per site per year (Ma and Bennetzen, 2004).

## Results and Discussion

**FIGURE 1:**
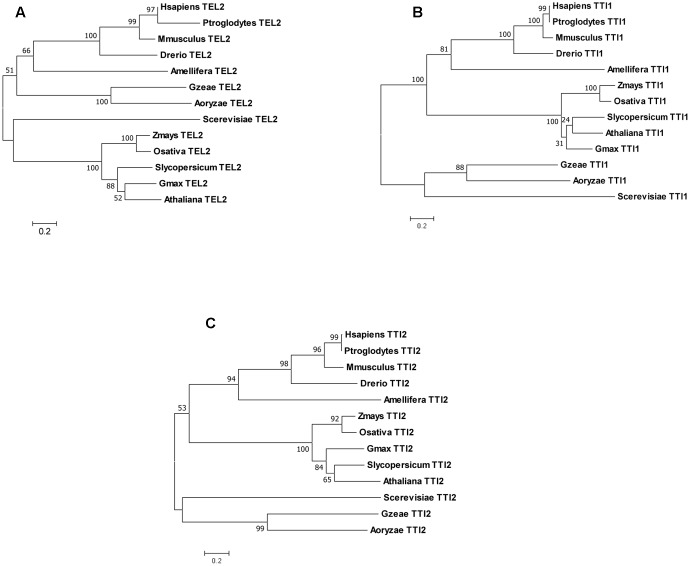
Phylogenetic analyses of TEL2 **(A)**, TTI1 **(B)**, and TTI2 **(C)** from animal, plant, and fungal species. The trees with the highest likelihoods are shown, with nodes indicating bootstrap support values (500 replicates). The bars below indicate distance scale in amino acid substitution per site.

**FIGURE 2:**
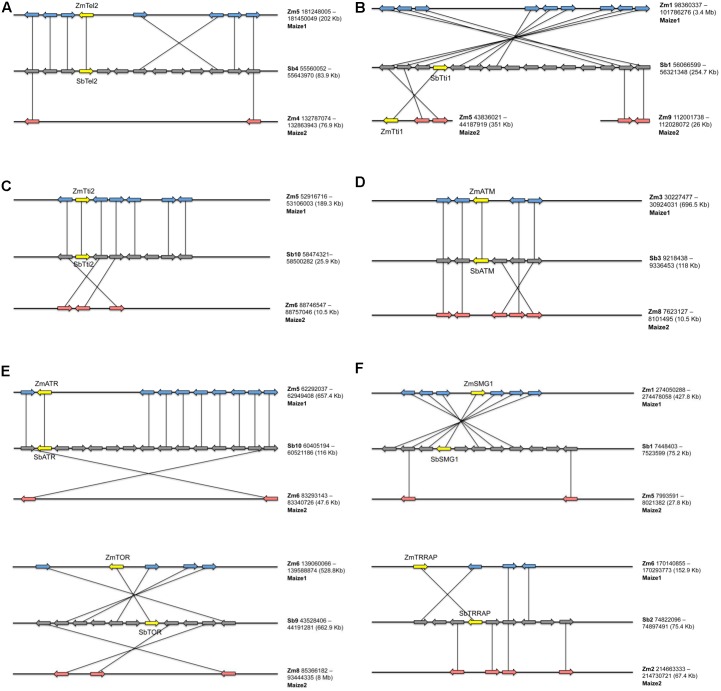
Synteny analysis of genes encoding members of TTT co-chaperone complex **(A–C)** and genes encoding their PIKK client proteins **(D–F)** in maize. Yellow genes indicate the copy that was retained after polyploidization in maize, and the orthologous gene in sorghum. Sorghum was used as a reference to align the maize syntenic regions (middle genomic segment with gray genes). The top (blue genes) and bottom (pink genes) genomic segments indicate syntenic regions from maize subgenomes 1 and 2 respectively. The coordinates and sizes of the syntenic regions are indicated to the right of the alignments.

Our analysis of the genomes of several animals, fungi, and plants indicate that *Tel2*, *Tti1*, and *Tti2* are single copy genes as they returned single BLASTP hits. Phylogenetic analyses of their sequences conform to the predicted evolutionary relationships between the species (**Figure 1**). Likewise, the PIKK genes in maize and rice are single copy (Supplementary Table 1) just like in *Arabidopsis* (Templeton and Moorhead, 2005) and humans (Bosotti et al., 2000). Synteny analysis in maize using sorghum as a reference confirmed that PIKK and TTT complex genes became single copy because of the removal of the duplicated copy from one of the homoeologs (**Figure 2**). Dataset from a previous study identified the two maize subgenomes as remnants of the two progenitors of maize, termed maize1 and maize2 (Schnable et al., 2011). In this study, it was hypothesized that fractionation in maize is based on a pattern of overexpression of genes of maize1 over the maize2 subgenome, referred to as genome dominance. It was further suggested that the copy from maize1 was favorably retained because it contributed more to total gene expression relative to its duplicate pair. However, here we can show that in case of the TTT complex *Tti1* is located on maize2, whereas the rest of the PIKK and TTT complex genes had copies retained on maize1. Such exceptions would indicate that selection for retention of a gene copy rather depends on the local pattern of transposition events than a particular subgenome. Indeed, the local chromosomal structural organization appeared to be required for the removal of gene copies because of historic homologous recombination events via unequal crossing over as shown for maize and foxtail millet compared to sorghum (Xu et al., 2012).

Therefore, it is not so much the dominance of one subgenome over the other one, but rather the mendelization of critical functions, which could explain the reversion to single copy of the genes investigated here. The interaction of TTT co-chaperones amongst themselves, as well as their interaction with PIKK proteins as co-chaperones, could be dosage-sensitive. Therefore, the genes coding for these proteins needed to evolve together as a unit (i.e., either all remain duplicated or all lose one duplicate) to attain the best stoichiometric balance needed to maintain fitness. However, it is also important to point out that none of the TTT complex and PIKK mutants displayed haplo-insufficiency, as heterozygotes seemed to be normal. It is therefore possible that expressions of these genes are subject to dosage compensation. If normal phenotypes in heterozygotes are conditional, they would display increased sensitivity compared to wild type, when subjected to certain levels of stress. For example, downregulation of *Tti2* expression in yeast is sufficient for normal growth. However, this downregulation strain was more sensitive to PIKK-related stresses compared to wild type (Hoffman et al., 2016). Another example is the *Steroidogenic factor-1* (*Sf-1*) gene in mouse wherein the heterozygote displayed mutant phenotypes only when subjected to stress (Bland et al., 2000). Aside from their role in protein folding, the TTT complex is also needed for the assembly of PIKK complexes (Horejsi et al., 2010; Kaizuka et al., 2010; Takai et al., 2010). Therefore, we extended our investigation to some components of TOR and ATR complexes, which were well-characterized in several organisms. Two proteins called RAPTOR (also known as KOG1) and LST8 (also known as GβL) are integral components of the TOR complex (Loewith et al., 2002; Kim et al., 2003). ATR, on the other hand, closely associates with ATRIP (LCD1 in yeast) (Cortez et al., 2001). Like the TTT complex and PIKKs, RAPTOR, LST8, and ATRIP are conserved in many eukaryotes and are also encoded by single copy genes (Cortez et al., 2001; Loewith et al., 2002). In addition, all three display haplo-insufficient phenotypes in yeast, which is evidence of their dosage-sensitive nature (Pir et al., 2012; Shimada et al., 2013). Homologs of these three genes have been cloned in *Arabidopsis* and in contrast to humans and yeast, both *RAPTOR* and *LST8* exist as two copies (Deprost et al., 2005; Moreau et al., 2012). However, their investigations revealed that one of the copies was barely expressed, and that mutation in the other copy is enough to cause a mutant phenotype. Moreover, disruption of the barely expressed copy of *RAPTOR* did not result in a mutant phenotype. Therefore, this is still consistent with the gene balance hypothesis. Analysis of many presence-absence variations (PAVs) in a diverse set of maize and teosinte lines suggests that fractionation might still occur in maize, and that it remains biased (Schnable et al., 2011). Possibly, epigenetic gene silencing is a prelude to the deletion of a gene copy and could reflect an ongoing fractionation in *Arabidopsis*.

**FIGURE 3:**
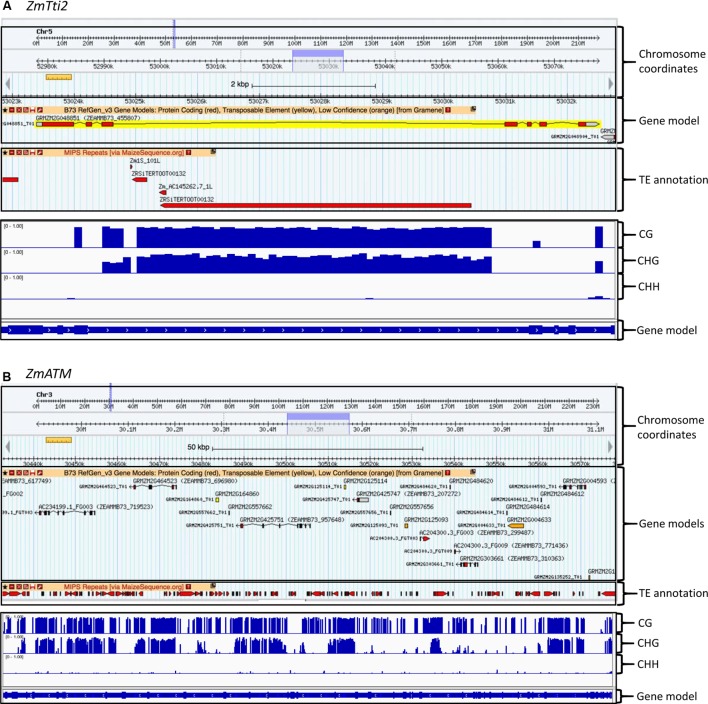
Examples of intronic TE insertions and gene body methylation found in some TTT complex and PIKK genes. The top panels show the TE insertion, while the bottom panels show DNA methylation in CG, CHG, and CHH contexts. **(A)** TE insertion and methylation in the third intron of *ZmTti2*. **(B)** Extensive TE insertions and methylation in many introns of *ZmATM*. Note that the *ZmATM* gene was misannotated and split into several gene models.

Given the potential critical dosage dependence of members of the TTT complex, their coding regions are sensitive to random transposon insertions that have taken place in maize, which also leads to epigenetic silencing. Based on a high confidence gene set, it was previously estimated that 11.6% of genes in maize contain TEs in their introns (Haberer et al., 2005). This estimate is close to the >10% estimate made by West et al. (2014) by surveying gene bodies for CHG DNA methylation and accompanying H3K9Me2 epigenetic modifications that mark the TEs. We found that six of the eight genes in our study contain TEs in introns (**Table 2** and Supplementary Figure 1). For example, there is a 6 Kb Gypsy retrotransposon in intron 3 of *ZmTti2*, and a 2 Kb hAT element in intron 12 of *ZmTti1*, which are absent in their putative sorghum orthologs. Estimation of the insertion times of five LTR retrotransposons indicated that three of them transposed less than 4.8 million years ago (Supplementary Table 2). This signifies that some of these TEs are recent transpositions that occurred after the split of maize and sorghum (Swigonova et al., 2004). The maize PIKKs *ZmATM*, *ZmATR*, *ZmTOR*, and *ZmSMG1* also have many TE insertions in their introns that expanded the gene size relative to their sorghum counterparts (**Table 2** and Supplementary Figure 1). For example, *ZmATM* genic region expanded to about 131 Kb relative to 71.5 Kb in its sorghum ortholog. However, this is not to imply that the TE insertions were selected for in these genes – they are most likely results of random transposon insertions. Because TEs are silenced by DNA methylation and associated histone modifications to prevent their expression (Slotkin and Martienssen, 2007), we investigated the methylation states of these TEs using datasets from a previous DNA methylation studies in maize (West et al., 2014). As expected, the TEs in the introns are heavily methylated in the CG and CHG contexts, as shown for *ZmTti2* and *ZmATM* in **Figure 3**. Nevertheless, these genes are still well-expressed in many tissues and at many times during development as shown by the gene expression database available in MaizeGDB. It has even been shown that insertion of a TE into an exon can permit proper gene expression as long as splicing is not affected (Wessler et al., 1987). To ensure that proper splicing occurred, we experimentally validated the expression of *ZmTti1* and *ZmTti2*, enabling us to clone their full-length coding sequences using RT-PCR (Garcia et al., 2017). All the data are indications of strong expression of these genes despite TE insertions in introns. In a genome-wide study, West et al. (2014) observed that about 10% of maize genes have CHG methylation in gene bodies that can be partially attributed retrotransposon insertions in the introns. The CG and CHG methylations are also mostly confined to introns where the TEs are located, indicating a mechanism to stop the spread of methylation to exons. This very accurate marking of boundaries between TE and genic sequences likely enable the genes to be expressed at the right dosage. Methylation in TEs promotes the formation of heterochromatin to suppress transposition (Cedar and Bergman, 2009; Zemach et al., 2013) and methylation in promoters is correlated with transcriptional repression (Weber and Schubeler, 2007). In contrast, the role of gene body methylation in the regulation of gene expression is still not resolved, although some studies found that gene body methylation is associated with transcriptional activation (Zhang et al., 2006; Wang et al., 2015), others dispute this (Bewick et al., 2016). Nevertheless, future studies in maize that probe the role of gene body methylation in gene expression, whether because of TE insertions in introns or not, will help determine whether it has a role in changing the expression level of a “connected” gene, and hence its potential impact on dosage-sensitive functions.

**Table 2:** Examples of TE insertions in introns of maize TTT complex and PIKK genes.

Maize gene	Intron TE insertion(s)	Maize gene length (Kb)	Sorghum ortholog gene length (Kb)
*ZmTti1*	2 Kb hobo DNA transposon	20.87	12.1
*ZmTti2*	6 Kb Gypsy LTR retrotransposon	9.1	4.2
*ZmATM*	2.2 Kb L1/CIN4 retrotransposon	131	71
	2.1 Kb L1/CIN4 retrotransposon		
	1.7 Kb L1/CIN4 retrotransposon		
	3.1 Kb Ty1/Copia retrotransposon		
	1.2 Kb Ty1/Copia retrotransposon		
*ZmATR*	2.4 Kb Gypsy LTR retrotransposon	45	31
	10.2 Kb Gypsy LTR retrotransposon		
	3 Kb Ty1/Copia retrotransposon		
	4.7 Kb Ty1/Copia retrotransposon		
*ZmSMG1*	1.4 Kb Ty1/Copia retrotransposon	30	22.6
	2.1 Kb L1/CIN4 retrotransposon		
*ZmTOR*	2 Kb L1/CIN4 retrotransposon	51	31.8
	5 Kb Ty1/Copia retrotransposon		


The reversion to single gene copy after genome duplication could be due to neutral loss of extra copies, or selection based on unbalanced gene duplications. It is likely that both mechanisms were involved in the diploidization of maize from its allotetraploid ancestor, which could be based on whether the genes belong to a dosage sensitive protein-protein network or not. Although our data on the TTT complex and PIKK genes is consistent with the gene balance hypothesis, the alternative hypothesis of neutral loss of extra copies cannot be discounted. To test this, uneven alterations in the gene copy number (or uneven levels of gene expression) should be done to see if it results in protein complex destabilization and thus potential negative fitness effects. This approach can be extended to other genes in maize to see if unbalanced duplications in members of protein complexes are deleterious in general.

## Author Contributions

NG designed the experiments, performed the analysis, and wrote the manuscript. JM designed the experiments and wrote the manuscript.

## Conflict of Interest Statement

The authors declare that the research was conducted in the absence of any commercial or financial relationships that could be construed as a potential conflict of interest.
