# Supplementary material for: TTT and PIKK Complex Genes Reverted to Single Copy Following Polyploidization and Retain Function Despite Massive Retrotransposition in Maize
Source: Front Plant Sci. 2017 Nov 7;8:1723. doi: 10.3389/fpls.2017.01723 (PMC5681926; doi:10.3389/fpls.2017.01723)
Supplement: Supplementary file 3 [file Presentation_1.PPTX]

## Slide 1
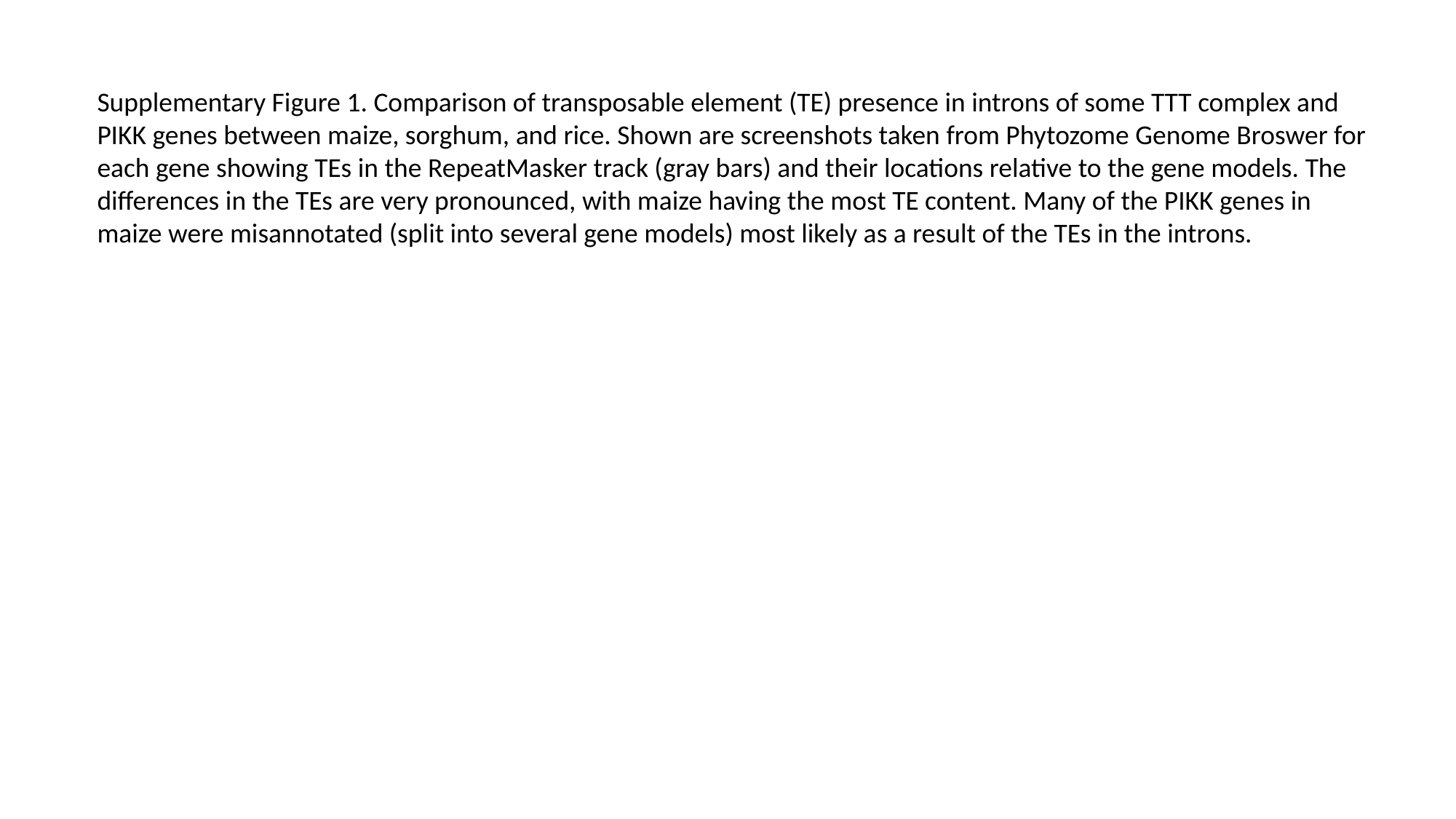

Supplementary Figure 1. Comparison of transposable element (TE) presence in introns of some TTT complex and PIKK genes between maize, sorghum, and rice. Shown are screenshots taken from Phytozome Genome Broswer for each gene showing TEs in the RepeatMasker track (gray bars) and their locations relative to the gene models. The differences in the TEs are very pronounced, with maize having the most TE content. Many of the PIKK genes in maize were misannotated (split into several gene models) most likely as a result of the TEs in the introns.

## Slide 2
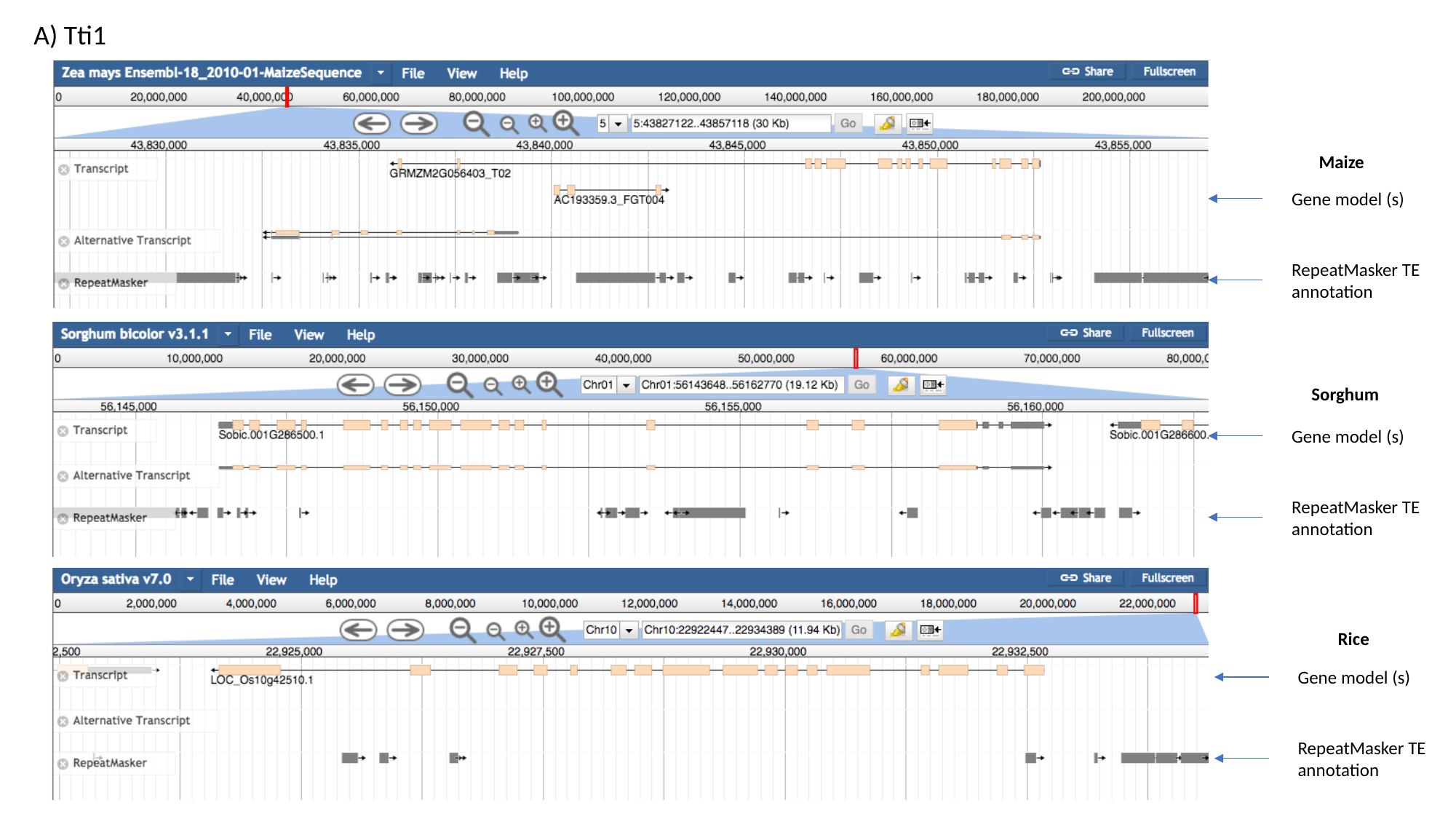

A) Tti1
Maize
Gene model (s)
RepeatMasker TE annotation
Sorghum
Gene model (s)
RepeatMasker TE annotation
Rice
Gene model (s)
RepeatMasker TE annotation

## Slide 3
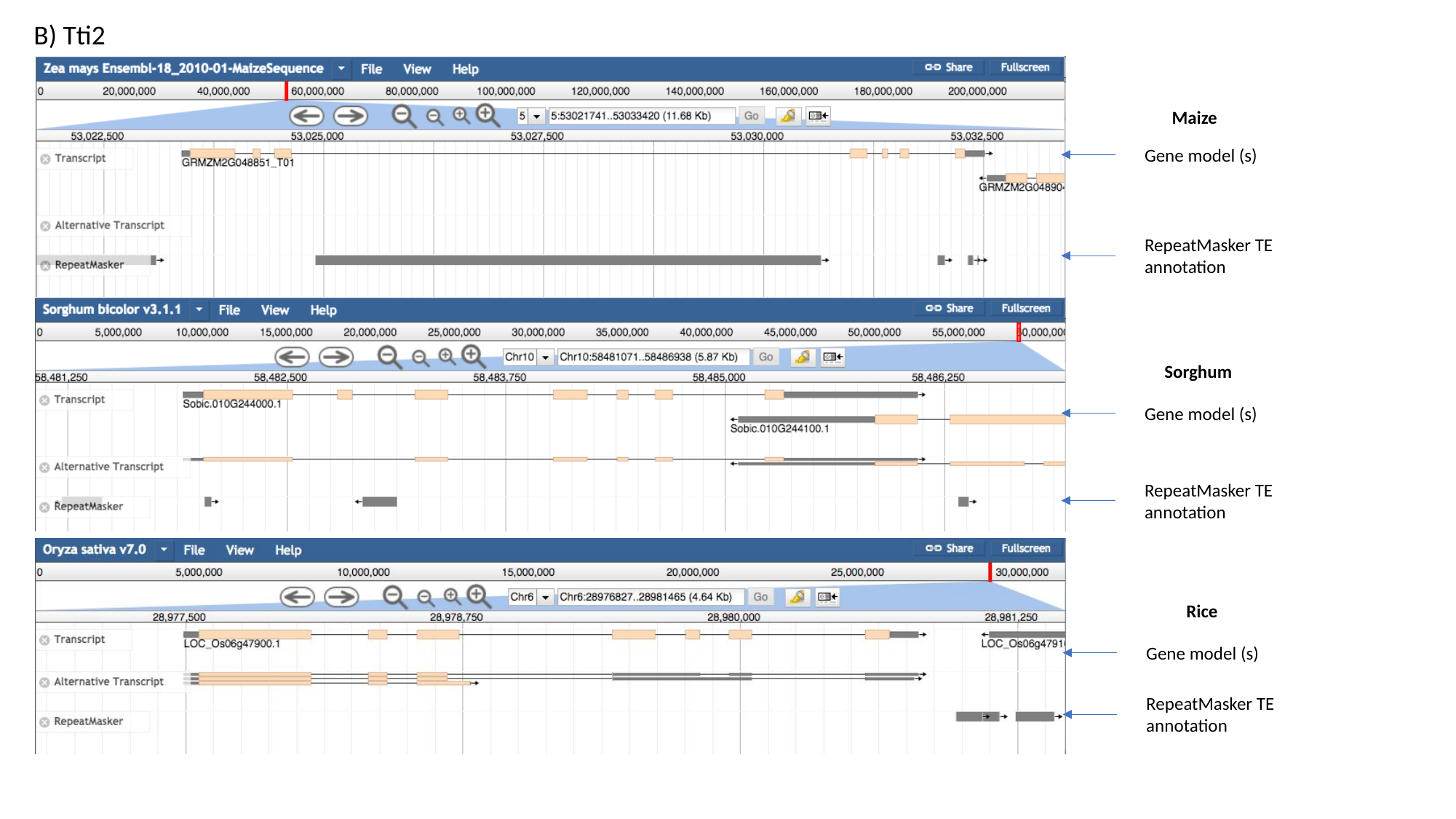

B) Tti2
Maize
Gene model (s)
RepeatMasker TE annotation
Sorghum
Gene model (s)
RepeatMasker TE annotation
Rice
Gene model (s)
RepeatMasker TE annotation

## Slide 4
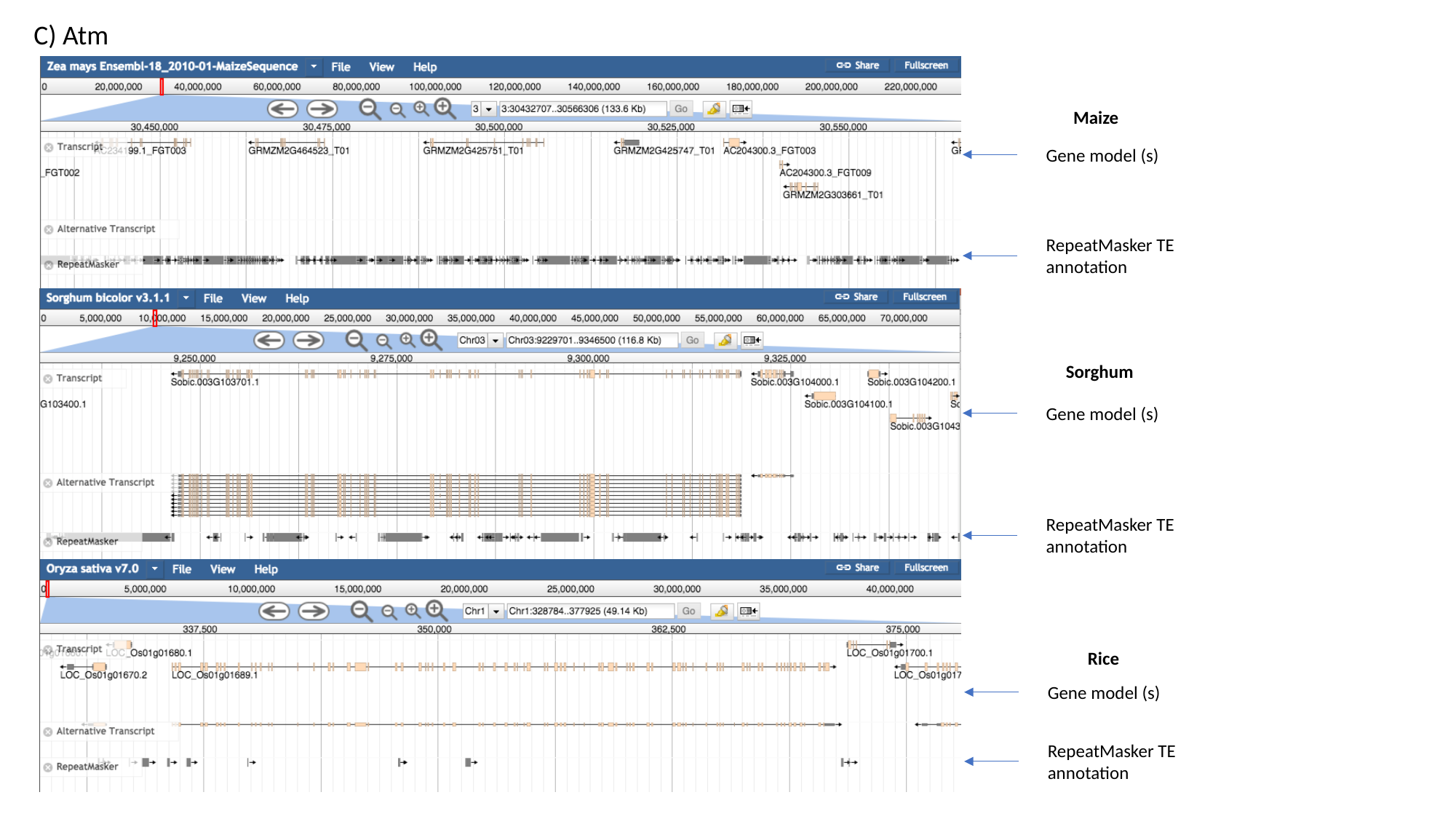

C) Atm
Maize
Gene model (s)
RepeatMasker TE annotation
Sorghum
Gene model (s)
RepeatMasker TE annotation
Rice
Gene model (s)
RepeatMasker TE annotation

## Slide 5
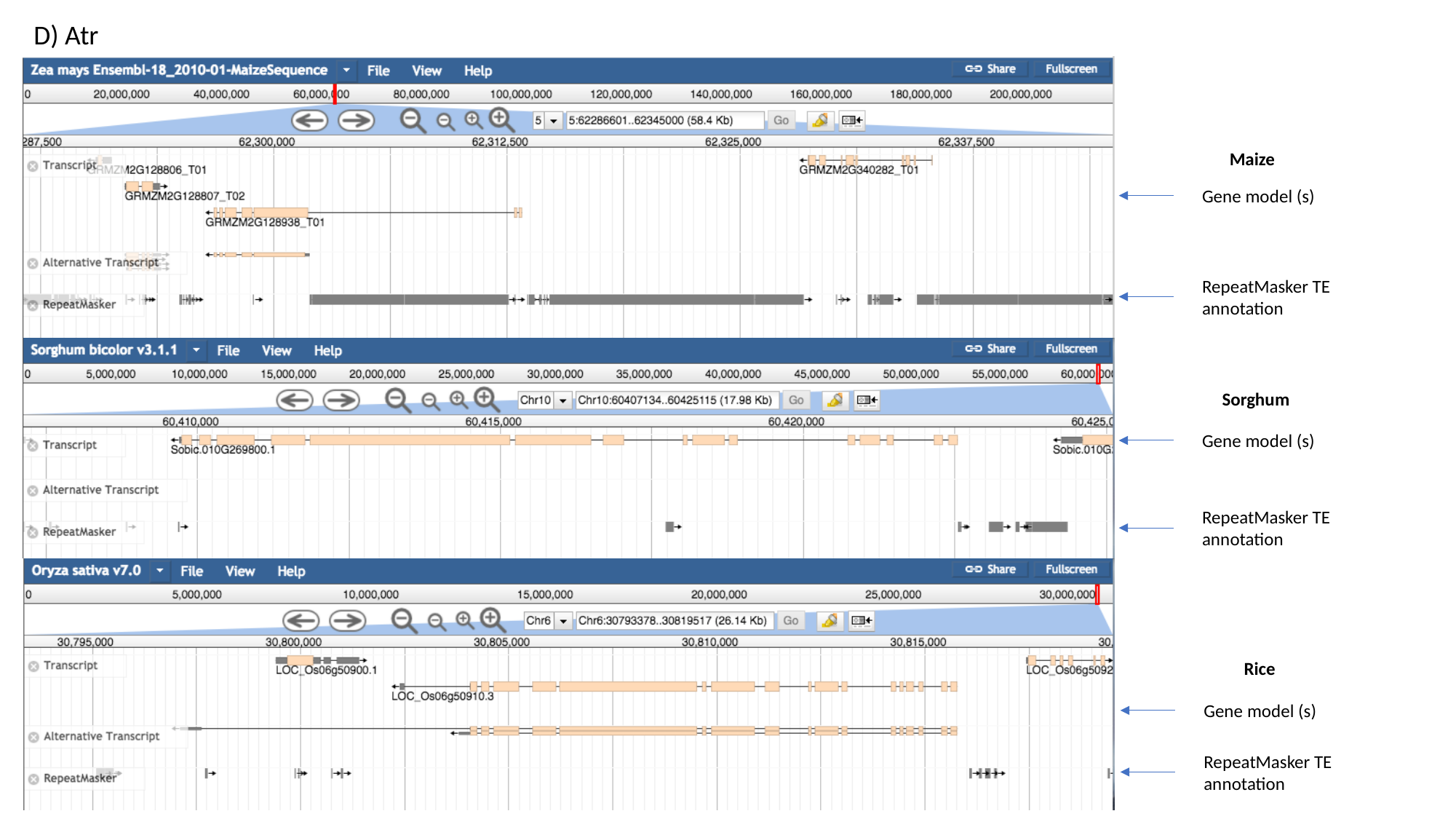

D) Atr
Maize
Gene model (s)
RepeatMasker TE annotation
Sorghum
Gene model (s)
RepeatMasker TE annotation
Rice
Gene model (s)
RepeatMasker TE annotation

## Slide 6
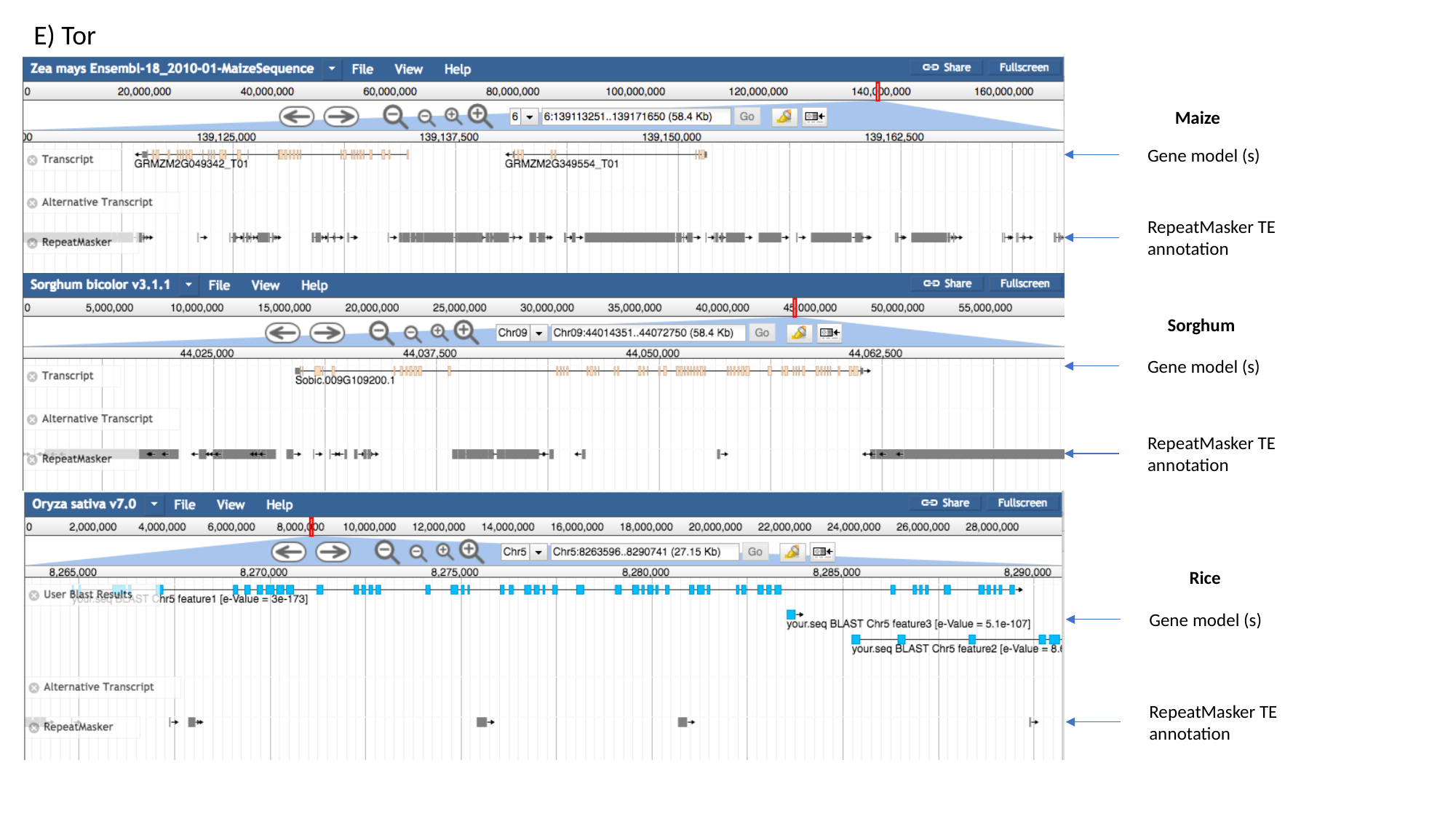

E) Tor
Maize
Gene model (s)
RepeatMasker TE annotation
Sorghum
Gene model (s)
RepeatMasker TE annotation
Rice
Gene model (s)
RepeatMasker TE annotation

## Slide 7
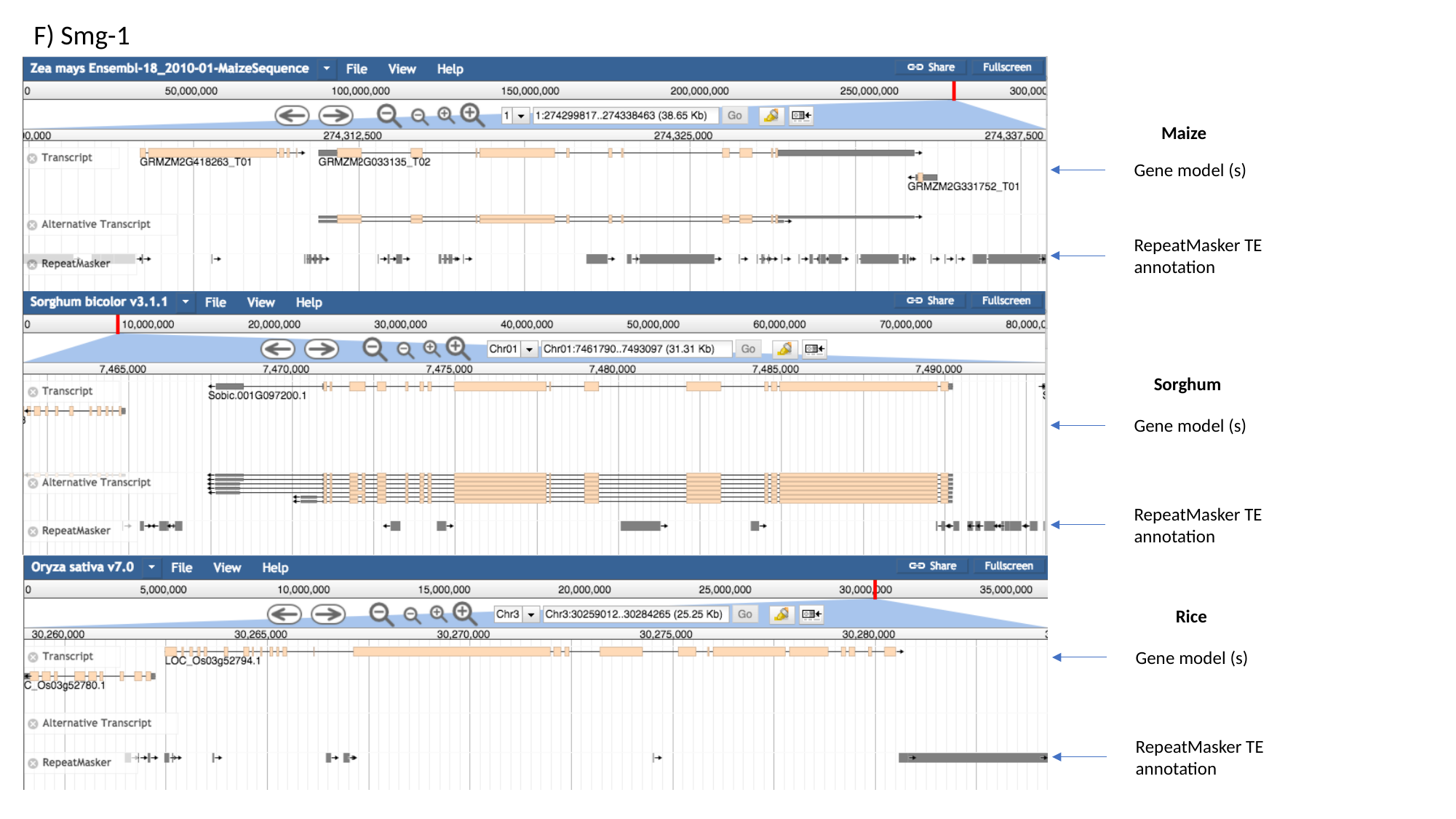

F) Smg-1
Maize
Gene model (s)
RepeatMasker TE annotation
Sorghum
Gene model (s)
RepeatMasker TE annotation
Rice
Gene model (s)
RepeatMasker TE annotation
